# Quantitative Assessment of the Impact of Blood Pulsation on Intraocular Pressure Measurement Results in Healthy Subjects

**DOI:** 10.1155/2017/9678041

**Published:** 2017-01-30

**Authors:** Robert Koprowski, Lei Tian

**Affiliations:** ^1^Department of Biomedical Computer Systems, University of Silesia, Faculty of Computer Science and Materials Science, Institute of Computer Science, Ul. Będzińska 39, 41-200 Sosnowiec, Poland; ^2^Beijing Institute of Ophthalmology, Beijing Tongren Eye Center, Beijing Tongren Hospital, Capital Medical University, Beijing Ophthalmology & Visual Sciences Key Laboratory, Beijing 100730, China

## Abstract

*Background*. Blood pulsation affects the results obtained using various medical devices in many different ways.* Method*. The paper proves the effect of blood pulsation on intraocular pressure measurements. Six measurements for each of the 10 healthy subjects were performed in various phases of blood pulsation. A total of 8400 corneal deformation images were recorded. The results of intraocular pressure measurements were related to the results of heartbeat phases measured with a pulse oximeter placed on the index finger of the subject's left hand.* Results*. The correlation between the heartbeat phase measured with a pulse oximeter and intraocular pressure is 0.69 ± 0.26 (*p* < 0.05). The phase shift calculated for the maximum correlation is equal to 60 ± 40° (*p* < 0.05). When the moment of measuring intraocular pressure with an air-puff tonometer is not synchronized, the changes in IOP for the analysed group of subjects can vary in the range of ±2.31 mmHg (*p* < 0.3).* Conclusions*. Blood pulsation has a statistically significant effect on the results of intraocular pressure measurement. For this reason, in modern ophthalmic devices, the measurement should be synchronized with the heartbeat phases. The paper proposes an additional method for synchronizing the time of pressure measurement with the blood pulsation phase.

## 1. Introduction

Today, it is difficult to imagine a physician of any specialization without complicated equipment designed to perform different types of measurements in patients. It would be difficult or even impossible to perform a contemporary diagnosis without a series of measurements. On the one hand, doctors have more and more confidence in the results obtained from medical devices. On the other hand, there often exist simple methods for obtaining more accurate results and reducing measurement errors. These methods take into consideration blood pulsation phases. The impact of this element on measurements is strongly dependent on the anatomical and functional links between the blood pulsation and the measured parameter [1]. One of these groups of methods and medical devices includes Diaton transpalpebral tonometer, Dynamic contour tonometer, Goldmann applanation tonometer, and noncontact tonometers: Ocular Response Analyzer (ORA), Corvis ST, for measuring intraocular pressure (IOP), and other additional parameters [2, 3]. Corvis ST, owing to the Ultra-High-Speed Scheimpflug camera, can record corneal deformation which is the response to an air puff. The change in the blood pulsation phase during measurement affects the results in different ways. Moreover, measurement errors occur which result from nonsynchronization of the measurement moment with the blood pulsation phase [4]. The effect of blood pulsation on the results obtained in IOP measurement is well known [5–7]. The authors of work [6], in particular, showed that the ocular pulse amplitude readings measured with dynamic contour tonometry in healthy subjects were not associated with blood pressure levels nor amplitude. It appears that the ocular pulse amplitude is strongly dependent on the time-course of the cardiac contraction. Both regulating mechanisms in the carotid system and scleral rigidity may be responsible for dampening the direct effect of blood pressure variations. Similarly the authors of work [7] confirmed, by means of experiments, that pulse amplitude, fundus pulsation amplitude, and pulsatile ocular blood flow depend on ocular volume and indicated that there is no reduction in the pulsatile component of ocular blood flow in the case of myopic patients. Accordingly, the relationship between axial eye length and pulsatile ocular blood flow seems to result from different ocular volumes. The research carried out by the authors in work [7] has important implications for the studies relating to pulse amplitude or pulsatile ocular blood flow. However, the dependence of the results obtained for a specific device, for example, the Corvis tonometer, is unknown. Currently, the Corvis tonometer allows for the measurement of a number of biomechanical characteristics of corneal deformation. These characteristics, whose dependence on the blood pulsation phase can vary, include the location of the first and second applanation, the size of flattening for the first and second applanation, the maximum corneal deformation, the maximum amplitude, and frequency of corneal vibration. On the other hand, the accuracy of measurement of these characteristics associated with the adopted image analysis and processing method is also vital. Various types of devices designed for detecting the corneal edge on a sequence of images from the Corvis tonometer can be applied here, ranging from Roberts, Sobel, or Prewitt filters to the Canny method or other profiled edge detection methods. The blood pulsation phase measurement itself can be also carried out with the use of various types of pulse oximeters corresponding to different types of absorption of radiation of two different wavelengths (red and infrared) by red blood cells in the capillaries. A variable component describes arterial blood absorbance and thus it is possible to calculate the degree of saturation of haemoglobin with oxygen. Therefore, the measurement of changes in the skin colour can be performed remotely without contact by means of laser light [8] or by analysing the skin image coming from the camera in visible light [9–11].

Considering all of the above factors, the analysis of the impact of blood pulsation phases on the results obtained from the Corvis tonometer is practically interesting. In particular, the following issues are of interest:

- confirmation of the dependence of IOP on the blood pulsation phase,
- quantitative assessment of the dependence of IOP on the blood pulsation phase,
- quantitative assessment of the dependence of 1st and 2nd applanation point, maximum corneal deformation, and amplitude of corneal vibrations for the frequency >100 Hz on the blood pulsation phase,
- indication of the scope of variation in IOP measurement for the lack of measurement synchronization,
- creation of new analysis methods for images and signals from the Corvis tonometer and pulse oximeter allowing for automatic and reproducible measurement of the above parameters.

The obtained results of synchronization of IOP measurement moment and other corneal deformation parameters with the blood pulsation phase are shown in this paper.

## 2. Material

Eight thousand and four hundred corneal deformation images of the eyes of 10 healthy subjects were analysed as part of the study. The subjects ranged in age from 27 to 36 years and women accounted for 66% of the whole study group. Six IOP measurements using the Corvis ST (software ver. 1.2, Oculus Optikgeräte GmbH, Wetzlar, Germany; resolution of 0.1 mmHg, measurement range from 1 to 60 mmHg) were performed for each eye (5 left and 5 right eyes) in different blood pulsation phases. For each series of 6 measurements per one patient, an attempt was made to carry them out evenly in the full range from 0° to 360° of the blood pulsation phase. A total of 60 results of IOP measurement were acquired (6 measurements for each of the 10 subjects). The exclusion criterion concerned patients who had ophthalmic surgery, heart surgery, hypertension, ocular hypertension, cardiac arrhythmias, bypass, and other diseases of the heart or were pregnant. A sequence of images of corneal deformation resulting from an air puff was acquired from the Corvis tonometer. The Ultra-High-Speed Scheimpflug camera, which is an integral part of the Corvis tonometer, enables us to register, within the time *I* = 140, images with a resolution *M* × *N* = 200 × 576 pixels. Each *i*th image was recorded every 231 *μ*s. The blood pulsation measurement was made with the Mindray PM-8000 Express (measurement range: from 0 to 100%, resolution of 1%, and pulse rate: from 20 to 254 beats/minute). The pulse oximeter was placed on the index finger of the subject's left hand. Multiple measurements of the same subject were carried out with minimum time intervals between successive measurements to prevent differentiation of the subject's pulse. All tests were carried out according to the Declaration of Helsinki on healthy subjects with their free and informed consent at the Beijing Tongren Hospital. The ophthalmologist during tests started the measurement (and at the same time triggered an air puff) which was recorded and saved together with the blood pulsation phase (in the data from the pulse oximeter). Further analysis of signals and images was carried out in software designed by the author in Matlab (Version 7.11.0.584, R2010b, Java VM Version: Java 1.6.0_17-b04 with Sun Microsystems Inc.) with Image Processing Toolbox (Version 7.1) and Signal Processing (Version 7.2) on a PC running Windows 7 Professional, 64-bit with the Intel Core i7-4960X CPU @ 3.60 GHz and HDD 3.5′′ SATA III 2 TB.

## 3. Method and Results

The measurement method proposed by the authors consists of two phases:

- analysis and processing of images from the Corvis tonometer, which enables us to calculate biomechanical characteristics of the deformed cornea and IOP,
- quantitative assessment of correlation between the blood pulsation phase and measured parameters (mainly IOP).

### 3.1. Analysis of Corneal Deformation Images

The analysis of corneal deformation images is related to an original method involving a series of operations performed on images from the Corvis tonometer. The input data, a sequence of images *L*_GRAY_(*m*, *n*) (where *m* is row and *n* is image column) with a resolution *M* × *N* = 200 × 576 pixels, are loaded into Matlab in *∗*.*jpg* or *∗*.*avi* format. Image preprocessing involves median filtering with a mask sized *M*_*h*_ × *N*_*h*_ = 3 × 3 pixels, the output image *L*_MED_(*m*, *n*). The filter mask size was selected based on the maximum size of a single noise (artefact) present in a 2D image *L*_GRAY_ that did not exceed 4 pixels. The next step is edge detection with the use of the Canny filter followed by joining the relevant parts of the contour. The issue of joining the edges entails a few problems associated with maintaining the corneal contour continuity. These problems include discontinuous edges and blank spaces between the detected contour portions. They are solved by using 5th-degree polynomial approximation. The polynomial degree was selected taking into account the anthropometric data of corneal curvature. Thus performed analysis for each *i*th image provides the image *L*_*C*_(*n*, *i*). On this basis, the corneal reaction *L*_*HC*_(*n*, *i*) is calculated (it is separated from the constant component, the corneal curvature at rest, and from the eyeball response). Based on preliminary studies and measurements, as well as biomechanical evidence and knowledge of ophthalmologists, the following features (which were measured) were selected:

*w*(1): 1st applanation point (momentary corneal flattening),
*w*(2): 2nd applanation point,
*w*(3): maximum corneal deformation,
*w*(4): frequency of corneal vibrations for the frequency >100 Hz,
*w*(5): intraocular pressure (read from the tonometer).

**Figure 1:**
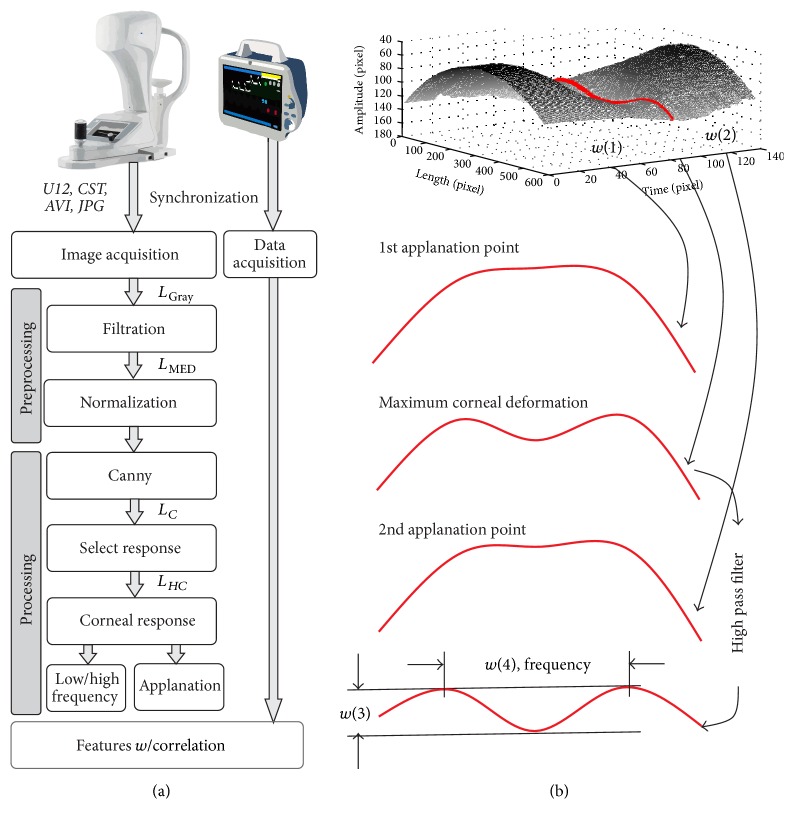
Block diagram of the idea of measuring the features *w* and the different phases of the analysis and processing of images from the Corvis tonometer (a) and the graph of corneal deformation for successive time sequences together with three characteristic waveforms (1st and 2nd applanation as well as the maximum corneal deformation) being the basis for calculating the features *w*(1), *w*(2), *w*(3), and *w*(4), respectively (b).

The block diagrams of the proposed image analysis and processing methods and feature measurement idea are shown in Figure 1. Examples of values calculated for the features *w* for different blood pulsation phases are shown in Table 1.

As is apparent from the presented table (Table 1), the smallest changes and the smallest values of standard deviation of the mean (std) are visible for the feature *w*(2) (the mean value of 21.54 ± 0.11 ms, ±0.51%, for a confidence interval *p* < 0.3) and for the other features: *w*(1): 7.47 ± 0.16 ms (±2.1%), *w*(3): 1.04 ± 0.03 mm (±2.8%), *w*(4): 416 ± 10.7 Hz (±2.5%), and *w*(5): 15.25 ± 0.61 mmHg (±4.0%) for *p* < 0.3 Student's *t*-distribution (an IOP unit was adopted in accordance with the units in tonometers). Detailed mean values of individual features for 10 subjects are shown in Table 2. The second measurement concerns correlation of the measured features *w* with the subject's pulse phase (feature *w*(6)).

### 3.2. Correlation between the Blood Pulsation Phase and Measured Parameters *w*

**Figure 2:**
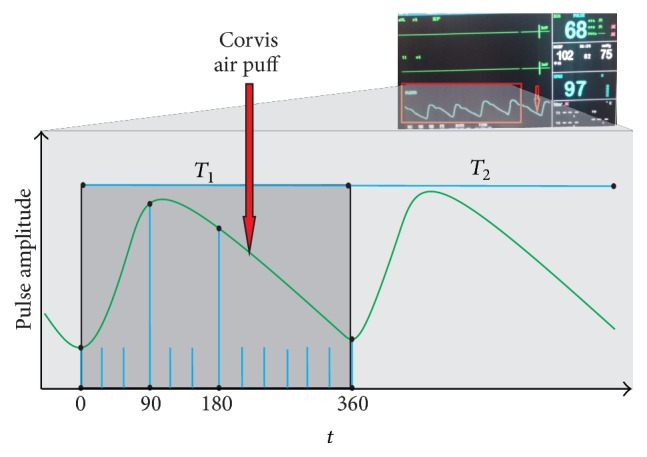
Graph of changes in the blood pulsation amplitude over time. A sample moment of starting the measurement with the Corvis tonometer and the conventionally adopted angular measurement scales are marked in red.

Blood pulsation measurement with the use of a pulse oximeter enables us to determine amplitudes of pulse changes with a resolution of 1%. The impact of blood pulsation (the phase impact) on corneal biomechanical measurements performed using the Corvis tonometer was measured on the index finger of the subject's left hand. This corresponds to the typical placement of the pulse oximeter during hospitalization or when testing patients.  Figure 2 shows the conventional coordinate system. The coordinate origin is the moment of measuring IOP for some of the 12 areas occurring every 30°. It means that the measurements were performed for 0°, 30°, 60°, 90°, and so forth. The adopted values and the analysis step result from simplification of analyses and measurement accuracy limitations (of the parameter and tonometer). The accuracy enables us to carry out a meaningful and repeatable measurement of the correlation for the same blood pulsation frequency. For 6 measurements performed for a single patient, the discrepancy in blood pulsation frequency was not more than 4%. The determined value of the blood pulsation phase was referred to as the feature *w*(6). From a practical point of view, it is important to indicate the correlation between the feature *w*(6) and the other features (from *w*(1) to *w*(5)). The correlation *r*_*v*_(*j*) was measured using the formula below where *k* is the number of the measurement for one subject (*k* ∈ (1,6)), *v* is the subject number (*v* ∈ (1,10)), and *j* is the feature number (*j* ∈ (1,5)); that is,

(1) rvj=∑k=1Kwk,vj−wk,vj−·wk,v6−wk,v6−∑k=1Kwk,vj−wk,vj−2·∑k=1Kwk,v6−wk,v6−2,

where *r*_*v*_(*j*) is correlation for *j*th feature and *v*th subject for *j* ∈ (1,5), *w*_*k*,*v*_(*j*) is *j*th feature of the *v*th subject of the *k*th measurement, and *K* is the total number of measurements.

**Figure 3:**
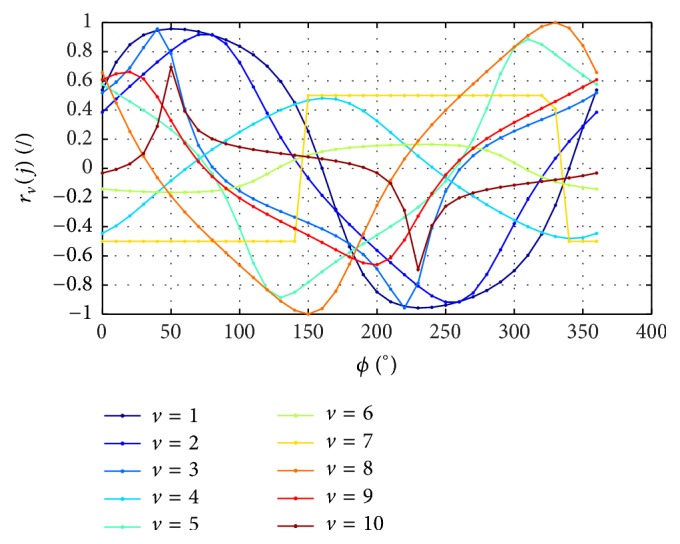
Graph of changes in the correlation *r*_*v*_(*j* = 5) of feature *w*_*k*,*v*_(*j* = 5) for individual subjects (*v* ∈ (1,10)) as a function of phase shift *ϕ*.

**Figure 4:**
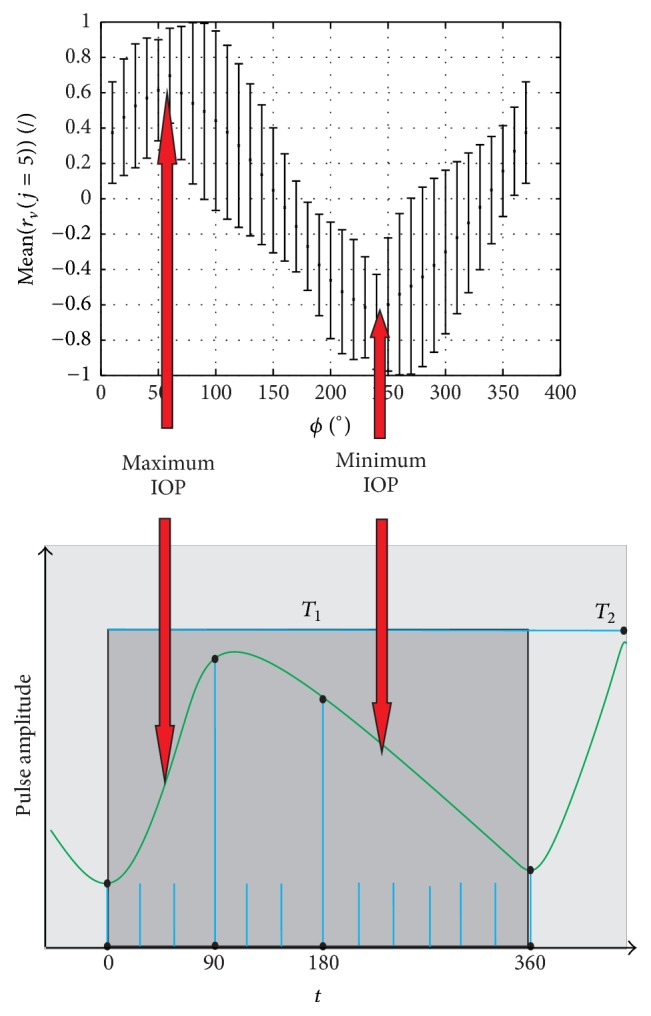
Graph of changes in mean correlation *r*_*v*_(*j* = 5) of the feature *w*_*k*,*v*_(*j* = 5) for individual subjects (after removing thick errors, outliers) as a function of phase shift *ϕ*. Additionally, the graph shows the position of the minimum and maximum IOP values relative to the blood pulsation phase. Minimum and maximum values of IOP are marked with red arrows. Blue lines on the *x*-axis indicate the arbitrarily adopted measurement times.

The natural phase shift in blood pulsation between the heart, the index finger (the measurement point), and the eye depends on the distance and subject's anatomical features. For the analysed data, correlation is determined according to formula (1) for subsequent, artificially added, phase shifts *ϕ* of the feature *w*_*k*,*v*_(*j*). The results of changes in correlation for the artificially added phase shifts *ϕ* are shown in Figure 3. The values of phase shift *ϕ* for which the correlation *r*_*v*_(*j*) reaches the maximum and minimum values are given in Table 3 for the feature *w*(5)  (*j* = 5). Further calculations were performed* under* the assumption of* the null hypothesis Ho* that there is a statistical relationship between the features *w*(1) to *w*(5) and *w*(6) and under the assumption of the alternative hypothesis *H*_1_ that this relationship does not exist. The calculated correlation for all the analysed subjects indicates statistical significance (*p* < 0.05 for Student's *t*-distribution) only for the feature *w*(5). Therefore, there is a significant correlation between the intraocular pressure and heartbeat phase (between the features *w*(5) and *w*(6)). For the measured group of subjects, this correlation is high (it is in the range from 0.48 to 0.99; the measurement for *v* = 6 was considered a thick error and rejected) and its mean value is 0.78 ± 0.19 (see Table 3). The results presented in Table 3 for the features *w*(5) and *w*(6) are extremely important in practice. Low values of mean std of changes in the features from *w*(1) to *w*(4) can here result from two elements. The first one is the limited resolution of the analysed image. For the image resolution *M* × *N* = 200 × 576 pixels, there is, on average, 20 *μ*m per one pixel. This means that the accuracy of measuring the feature *w*(1) as well as features *w*(2), *w*(3), and *w*(4) is limited to the resolution of ±20 *μ*m. So, if, for example, the amplitude for the first applanation changes by less than 20 *μ*m for the next *i* frames (images in a sequence), the measurement error of the feature *w*(1) will be 231 *μ*s. The other element influencing the low values of mean std of changes in the features from *w*(1) to *w*(4) is the lack of correlation with the heartbeat phases. For subjects *v* = {1,2, 3,4, 5,7, 8,9, 10}, there was a close relationship between the heartbeat phase and IOP. According to the diagram shown in Figure 3, the highest values of IOP are obtained for the phase shift of 60°. Minimum IOP values are obtained for the phase shift of 240° (60° + 180°); see Figure 4. The correlation for these angular values is 0.69 ± 0.26 (*p* < 0.05). The changes in mean correlation *r*_*v*_(*j* = 5) for individual subjects (after removing thick errors, outliers) as a function of phase shift *ϕ* presented in Figure 4 clearly indicate a strong correlation (0.69 ± 0.26) between IOP and the heartbeat phase.

## 4. Discussion

The impact of blood pulsation and its phases on measurements performed in medicine is known primarily from electrocardiogram (ECG). A lot of interesting publications have been written in this area [12–26]. In the first one [16] related to continuous cuffless blood pressure estimation using the pulse transit time and photoplethysmogram intensity ratio, the authors presented an algorithm which was validated on 27 healthy subjects with continuous Finapres blood pressure as a reference. The results showed that the mean std for the estimated systolic, diastolic, and mean blood pressure with the proposed method against reference was −0.37 and 5.21 mmHg, −0.08 and 4.06 mmHg, and −0.18 and 4.13 mmHg, and the mean absolute differences were 4.09 mmHg, 3.18 mmHg, and 3.18 mmHg, respectively. In the next work [20], the authors referred to the pulse transit time as a predictor of the efficacy of a celiac plexus block in patients suffering from chronic intractable abdominal pain. A celiac plexus block was successful in 9 out of 12 cases; the pulse transit time of the success group was 6.84 ± 5.04% versus 0.72 ± 0.78% in the failure group (*p* = 0.021). In turn, in work [14], the pulse transit time was calculated for each ECG R-wave and the corresponding steepest upstroke slope in the photoplethysmogram and was transformed to a continuous blood pressure estimate using multipoint nonlinear regression calibration based on the individual subject's sphygmomanometer readings. Bland-Altman limits of agreement between pulse transit time-derived systolic blood pressure estimates and sphygmomanometer values were −24.7 to 24.1 mmHg and between Portapres and sphygmomanometer systolic blood pressure values were −42.0 to 70.1 mmHg. For beat-to-beat systolic blood pressure estimation during exercise, pulse transmit measurement combined with multipoint nonlinear regression calibration based on intermittent sphygmomanometry can constitute an alternative to volume clamp devices. In work [17], the authors showed that the PTT-based blood pressure estimation may not be accurate enough since the regulation of blood pressure within the human body is a complex, multivariate physiological process. Taking into consideration the negative feedback mechanism in the blood pressure control, the authors introduced the heart rate (HR) and the blood pressure estimate in the previous step to obtain the current estimate. They validated this method by using the clinical database. Authors' results show that the pulse transit time, HR, and previous estimate reduce the estimated error significantly when compared to the conventional pulse transit time estimation approach (*p* < 0.05). There are also other interesting solutions relating to, for example, a wearable vital signs monitor at the ear [18], measuring short-term blood pressure variability: a comparison with the Finometer [23], comparison of ubiquitous blood pressure monitoring via pulse transit time [25], and others [12, 15, 19, 21]. Decision trees [26] or a new measurement system BioWatch described in work [13] were also used in the beat-to-beat and phase shift analysis. However, the authors of the above-mentioned publications did not make any reference to the analysis of the impact of blood pulsation phase on measurements in ophthalmology, in particular, measurements that are carried out using small and also relatively affordable devices (such as Corvis).

In the group of publications devoted to image analysis and processing, there are a lot of interesting works on ophthalmology, starting with those on the biomechanics of the eye associated with the Corvis tonometer [27–30], which show the effect of keratoconus, glaucoma, or diabetes on the results obtained, and ending with those on the analysis and processing of corneal edge images [31–35], which examine various methods of edge detection and measurement of additional biomechanical parameters. One of the few approaches to analysis of blood pulsation presented in the literature and related to the eye concerns the measurement of pupil size changes resulting from blood pulsation [36]. However, none of the presented methods of both image analysis and analysis of results takes into account the impact of blood pulsation on the obtained results of biomechanics of the eye (including IOP), which is presented in this paper. It should be emphasized that the presented image analysis and processing methods are one possible solution to this problem. There are also approaches described in the literature which are based on a fast Fourier transform (FFT) [37–41], computational intelligence systems [42–44], or fuzzy algorithms, used in other fields of life (for other applications).

Blood pulsation in ophthalmology is generally known (for other devices than Corvis) and its influence on measurement results is still under examination. It should be specifically noted that the impact of parameter changes on the results obtained is of high clinical importance. In the case of the impact of blood pulsation or the effect of other parameters (such as patient positioning during examination), different measurement results are obtained (in this case IOP). The so-called sensitivity to changing parameters enables us to clarify and define medical procedures. It indicates to what extent a given parameter may distort the result. Moreover, it indicates how the parameters such as patient positioning, head position, or, as in the discussed case, blood pulsation affect (quantitatively) the results. According to the authors, these procedures should be carried out for each medical device. Usefulness and reliability of such procedures should be verified both with ophthalmologists and with technicians operating the devices used in ophthalmology. Therefore, the issues described in this paper are current and important for both engineering and practical diagnostic usefulness in everyday work of ophthalmologists.

## 5. Conclusions

The study confirmed the correlation between the heartbeat phase and IOP measurement result. This correlation is high and amounts to 0.69 ± 0.26 (*p* < 0.05). The phase shift for the analysed cases (after 6 measurements per subject using the pulse oximeter placed on the index finger of the left hand) is 60 ± 40° (*p* < 0.05) for the maximum correlation. In the range of minimum and maximum correlation values, the mean std of IOP changes is even ±2.31 mmHg (*p* < 0.3). It should be noted that the sources of errors include alternating rhythm (pulse) of the heart. For the performed measurements, heart rate variability was in the range of 4% (typically 84 beats/minute). Therefore, it was at the level of other errors resulting from the finite image resolution and algorithm errors (range of ±1 pixel when detecting the corneal contour during its deformation). In summary, the paper

1. proposes a reproducible and fully automated method for measuring the features *w* based on the sequence of images obtained from the Corvis tonometer;
2. indicates which features *w* are dependent on the blood pulsation phase, in particular feature *w*(5);
3. confirms the dependence of IOP on the blood pulsation phase for the Corvis tonometer;
4. shows the reproducibility of the method for the discussed subjects.

The authors in subsequent works intend to carry out further studies on the impact of heartbeat rate changes or the anatomy of patients on the results of IOP and repeatability of measurements. In particular, the relationship between individual anatomical characteristics of patients and the obtained phase shift values will be explored. The presence of correlation between the heartbeat phase and IOP confirmed in this study led the authors to propose a system for connecting heartbeat phase measurement with triggering the measurement in the Corvis tonometer. In its current form, the heartbeat measurement is carried out using a CCD camera which is placed on the forehead support of the Corvis tonometer. The camera records changes in absorption of red radiation by red blood cells in the capillaries. The camera complements the modified system for measuring intraocular pressure patented by the author [37]. Currently, the method is being tested at the Beijing Institute of Ophthalmology, Beijing Tongren Hospital in China.

## Figures and Tables

**Table 1:** Examples of measured values of features *w* (obtained from the Corvis tonometer) for a single healthy subject, for one right eye (*v* = 1), and 6 measurements.

Measurement number	*w*(1) [ms]	*w*(2) [ms]	*w*(3) [mm]	*w*(4) [Hz]	*w*(5) [mmHg]
1	7.363	21.444	1.015	432	15
2	7.331	21.646	1.039	421	14.5
3	7.64	21.468	1.091	407	15
4	7.693	21.672	1.069	409	16
5	7.365	21.574	1.02	420	15
6	7.46	21.394	1.024	432	16

**Table 2:** Mean values of individual features *w* for 10 subjects (*p* < 0.3).

	Eye	*w*(1)	*w*(2)	*w*(3)	*w*(4)	*w*(5)
*v* = 1	Right	7.41 ± 0.15	21.54 ± 0.11	1.03 ± 0.03	416 ± 1.7	15.25 ± 0.6
*v* = 2	Right	7.38 ± 0.22	21.86 ± 0.51	1.04 ± 0.03	430 ± 3.1	14.9 ± 2.31
*v* = 3	Right	7.25 ± 0.32	22.05 ± 0.09	1.08 ± 0.033	444 ± 3.7	12.41 ± 0.49
*v* = 4	Right	7.30 ± 0.11	21.78 ± 0.18	1.08 ± 0.04	401 ± 2.0	14.33 ± 1.08
*v* = 5	Right	6.61 ± 0.08	22.81 ± 0.15	1.17 ± 0.03	399 ± 4.7	7.9 ± 0.63
*v* = 6	Left	6.71 ± 0.06	22.63 ± 0.09	1.11 ± 0.02	409 ± 3.1	8.08 ± 0.73
*v* = 7	Left	6.91 ± 0.03	22.13 ± 0.05	1.15 ± 0.01	434 ± 5.1	10.08 ± 0.28
*v* = 8	Left	6.91 ± 0.06	22.13 ± 0.15	1.16 ± 0.02	421 ± 1.4	10.08 ± 0.76
*v* = 9	Left	7.29 ± 0.13	21.94 ± 0.3	1.03 ± 0.045	409 ± 2.2	13.9 ± 1.5
*v* = 10	Left	7.09 ± 011	22.43 ± 0.2	1.15 ± 0.06	389 ± 3.7	12.1 ± 1.08

**Table 3:** Results of the minimum and maximum correlation between the features *w*(5) and *w*(6) for 10 subjects (outliers are in italic font).

	min⁡(*r*_*v*_(*j* = 5))	*ϕ*	max⁡(*r*_*v*_(*j* = 5))	*ϕ*
*v* = 1	−0.95	230	0.95	50
*v* = 2	−0.91	250	0.91	70
*v* = 3	−0.95	220	0.95	40
*v* = 4	*−0.48*	*340*	*0.48*	*160*
*v* = 5	−0.88	130	0.88	310
*v* = 6	*−0.16*	*50*	*0.16*	*230*
*v* = 7	*−0.51*	*100*	*0.5*	*160*
*v* = 8	−0.99	150	0.99	330
*v* = 9	−0.66	200	0.66	20
*v* = 10	−069	230	0.69	50
